# Improving the performance of fluidized bed reactor using rotating distributor and intelligent modeling

**DOI:** 10.1038/s41598-026-42831-2

**Published:** 2026-03-26

**Authors:** Hamada Mohmed Abdelmotalib

**Affiliations:** Dept. of Mechanical Power and Energy Engineering, Faculty of Engineering, Minia University, Minia, 61111 Egypt

**Keywords:** Energy science and technology, Engineering

## Abstract

A swirling fluidized bed reactor (SFBR) has been used as a promising technique for renewable energy applications, such as biomass combustion, gasification, and pyrolysis systems, due to its superior solid-gas thermal efficiency and mixing. This study presents an innovative approach to improve SFBR performance through the use of a rotating annular blade air distributor, considering the limitations of traditional fluidized beds. This study also provides a novel hybrid artificial intelligence (AI) model for further optimization and predicting the complex multiphase flow and heat transfer processes in SFBRs. The hybrid model integrates an artificial neural network (ANN) with particle swarm optimization (PSO) and is trained on a comprehensive experimental dataset across a range of operating conditions, including inlet gas velocity, distributor rotational speed, and radial and axial positions of the reactors. The study findings highlight that using a rotating distributor significantly enhances reactor performance, as increasing the rotational speed of the distributor decreased the bed pressure drop by 20% and increased the heat transfer coefficient by up to 30%. Furthermore, the proposed ANN–PSO model can provide an interpretable, fast, and efficient approach to predict and model the multiphase flow in SFBRs with high accuracy (R² = 0.991 for heat transfer). The study findings provide a foundation for future investigations on fluidized bed technologies for energy applications.

## Introduction

Fluidized bed reactor (FBR) is an effective and environmentally friendly technology for burning both conventional and renewable solid fuels for energy generation^1^. Among the types of FBR, swirling fluidized bed reactors (SFBR) have been recently recommended as one of the most attractive fluidized bed technologies^2^. The swirling fluidized bed is used to enhance solid-gas contact and solve some problems found in conventional fluidized beds, such as channeling and slugging, by introducing tangential motion using a blade air distributor^3^. The tangential motion improves the particle transfer mixing process and minimizes the elutriation of particles, which results in enhanced mass and heat transfer processes^4^. The swirling fluidized bed reactor, SFBR, exhibits many advantages that are confirmed by experimental results, such as better particulate mixing, significant enhancement of fluidization quality, removal of particle aggregation, decreased elutriation and operating costs, and minimized defluidized regimes^5–8^. There are several distributors designed to overcome the drawbacks of conventional FBR, such as swirling FBR and rotating FBR with static geometry. However, studies relevant to these types are very limited compared to those related to conventional FBR. The solid-gas flow, wall wear, and mixing behavior of particles were studied in SFB with an air plenum^9^. Increasing velocity resulted in the formation of different operating regimes. In stable swirling regimes, particles are subjected to attachment to the wall. The swirling velocity and bed pressure drop decreased with a reduction in the height of the central body. The heat transfer process in SFB was numerically studied^10^ temperature fields and solid holdup were predicted. The study results indicated that increasing the operating velocity improved the binary mixture behavior and the heat transfer process. Changing the blade inclination angle from 45° to 12° resulted in an enhancement of the heat transfer coefficient by 20%. The influences of using an annular spiral air distributor on the hydrodynamics, combustion, and emission characteristics of a cold model swirling fluidized-bed combustor (SFBC) for variable sand bed particle sizes and static bed heights were experimentally studied^11^. Four operational regimes of the bed were exhibited depending on the superficial air velocity. Optimal bed characteristics (sand particle size and bed height) and the range of primary air were determined before the combustion tests based on the results from the ‘cold’ hydrodynamic study. Three different distributor designs: perforated plates, circular-edged slots (90º), and novel swirling (45º) distributors were used^5^ to investigate the enhancement of mixing of bed materials in a fluidized bed. Particulate alumina grit and river sand (Geldart B) were used. The results indicated that the novel distributor with 45º inclined slots adequately enhances the circulation rate and mixing process. The perforated distributor was made possible by a novel swirling-type distributor without the implementation of an electric motor and mechanical rotation. Uniform and non-uniform nozzle-grid gas distributors were used^12^ to investigate wall-to-bed heat transfer at a tube bundle heat exchanger immersed in a bubbling bed of Geldart B particles. The non-uniform nozzle-grid gas distributor was used to overcome the increase in heat transfer coefficient in the core compared to the wall when using a uniform nozzle-grid gas distributor. The results indicated that the heat transfer process was enhanced in the case of using the modified gas distributor. Using machine learning approaches has recently been adopted as an alternative tool to numerous experimental methods to investigate and analyze large amounts of complex data in a limited time, distinguishing and estimating the relationship between input parameters and measured results without the need to conduct actual experiments^13^. The machine learning method was used to estimate the effect of process parameters on the fluidization characteristics^14^. The results illustrated that the radial position had the highest effect on the segregation of species and local mass flux, while the mass flux was the most dominant parameter for the local concentration of bed particles. A CFD-DNN hybrid model was employed to evaluate and predict the hydrodynamics of the fluidized bed^15^. Different hydrodynamic parameters were investigated, including bed pressure drop, solid velocity field, and solid volume fraction. Integrating the DNN model with CFD had superior spatial learning abilities, decreasing the required computational time and cost without affect the results accuracy^16^. A novel hybrid model was created to optimize real-time control of the methanol to olefin process in a fluidized bed. The developed hybrid model could predict the outlet variables of the reactor with RMSE, MRE, and solution time lower than 2.8, 7.63%, and 3 s, respectively. This model is proposed to offer new insights for other reaction processes. The ANN model was used to study the heat transfer in the freeboard of a bubbling fluidized bed combustor^17^. The combustor performance was analyzed using a backpropagation algorithm composed of 10 hidden layers. The chamber height was the most important variable affecting heat transfer in the combustion chamber, while the effects of ash content and enthalpy of flue gas were 8.6% and 4%, respectively. The analyses using the proposed multilayer model can be achieved for different input data with high accuracy. The drying behavior of a column bed dryer was predicted using the ANN model^18^. The proposed model was developed with different parameters such as sampling type, split ratio, epoch and number of neurons, and activation function. The model achieved the best prediction with a split ratio of 0.85, an activation function of rectifier, a number of neurons of 50 and 90 for the first and second layers, and an epoch number of 1800. The AI model was used to optimize the pyrolysis of CH_4_ in a large-scale fluidized bed reactor^19^. The obtained results can be used in the optimization and effective design of H_2_ production plants with low computational costs and high accuracy. The ANN model was employed to model the gasification of tyre in a bubbling fluidized bed reactor with different types and algorithms^20^. Among all ANN models were used, the generalized regression neural network achieved the best performance. The developed ANN model provides essential insights for tyre waste conversion, determining the optimal strategies for the gasification process and operational parameters. To improve the production of hydrogen in large-scale fixed and fluidized bed reactors, both CFD and machine learning techniques were integrated^21^. The CFD data was used to develop the machine learning model, which could predict the dry methane reforming process, enabling the analyze of the performance of the reactor and estimating the efficient method to increase the production of hydrogen. Swirling fluidized bed reactors (SFBRs) have been used as an alternative to conventional fluidized beds, providing excellent gas–solid contact, high rates of heat and mass transfer, and uniform distribution of temperature. The use of a rotating distributor is a key renovation driving these enhancements, which promotes particle dynamics and introduces a swirl flow within the bed. Using the rotating distributor adds extra complexity to the flow structure, increasing the difficulty of predicting and modeling the reactor performance of these reactors using traditional computational fluid dynamics simulations or traditional empirical models alone. In addition to the limitations of experimental techniques to specific configurations and the failure to determine the full nonlinear interactions between operating parameters. To address these limitations, this work introduces a novel AI hybrid model, that integrates the obtained experimental data with an artificial neural network (ANN) trained using particle swarm optimization (PSO). The proposed model enables accurate prediction of bed pressure drop and heat transfer coefficients across different operating conditions, with particular concern for the effect of swirl intensity addressed by the rotating distributor. The AI model not only emphasizes the strong agreement with experimental results but also presents interpretability through variable importance analysis and visualization of thermal and flow fields. The integrated AI-experimental approach contributes a new tool for optimizing and understanding the performance of swirling fluidized reactors employing dynamic distribution techniques.

## Materials and methods

### Materials

In this study, two materials were used in a gas-solid SFBR: air was used as the primary phase, and sand was used as the secondary phase. Sand (Geldart B type)^22^ with an average size of 1.1 mm and a static head of 7 cm was used as the bed material, fluidized by air at different air velocities. Geldart type B is widely used in industrial fluidized bed applications due to its resistance to elutriation and unique fluidization characteristics. A sieve analysis was employed to calculate the average size of bed particles. Mechanical sieving was used to measure the average size to sample of 500 g of sand using standard sieves ranging from 0.2 mm to 2 mm. The retained mass on each sieve was recorded, and then the accumulated distribution of particle size was determined. The analysis results indicate that the particles are predominantly in the range of 0.95 mm to 1.30 mm, with an average particle diameter of 1.1 mm. This provides a uniformity index of around 1.32, suggesting a relatively uniform distribution of particle size, which improves the fluidization quality. The properties of air and sand particles are given in Table 1.

**Table 1:** The properties of air and sand particles used in the experiments.

Property	Air	Sand
Diameter (mm)	-	1.1
Density (kg/m^3^)	1.2	2600
Thermal conductivity (W/m K)	0.025	0.25
Specific heat (J/kg K)	1005	820
Volume fractions	0.4	0.6
Sphericity	0.9	-
Minimum fluidization velocity (m/s)	0.8	

### Experimental setup

**Fig. 1:**
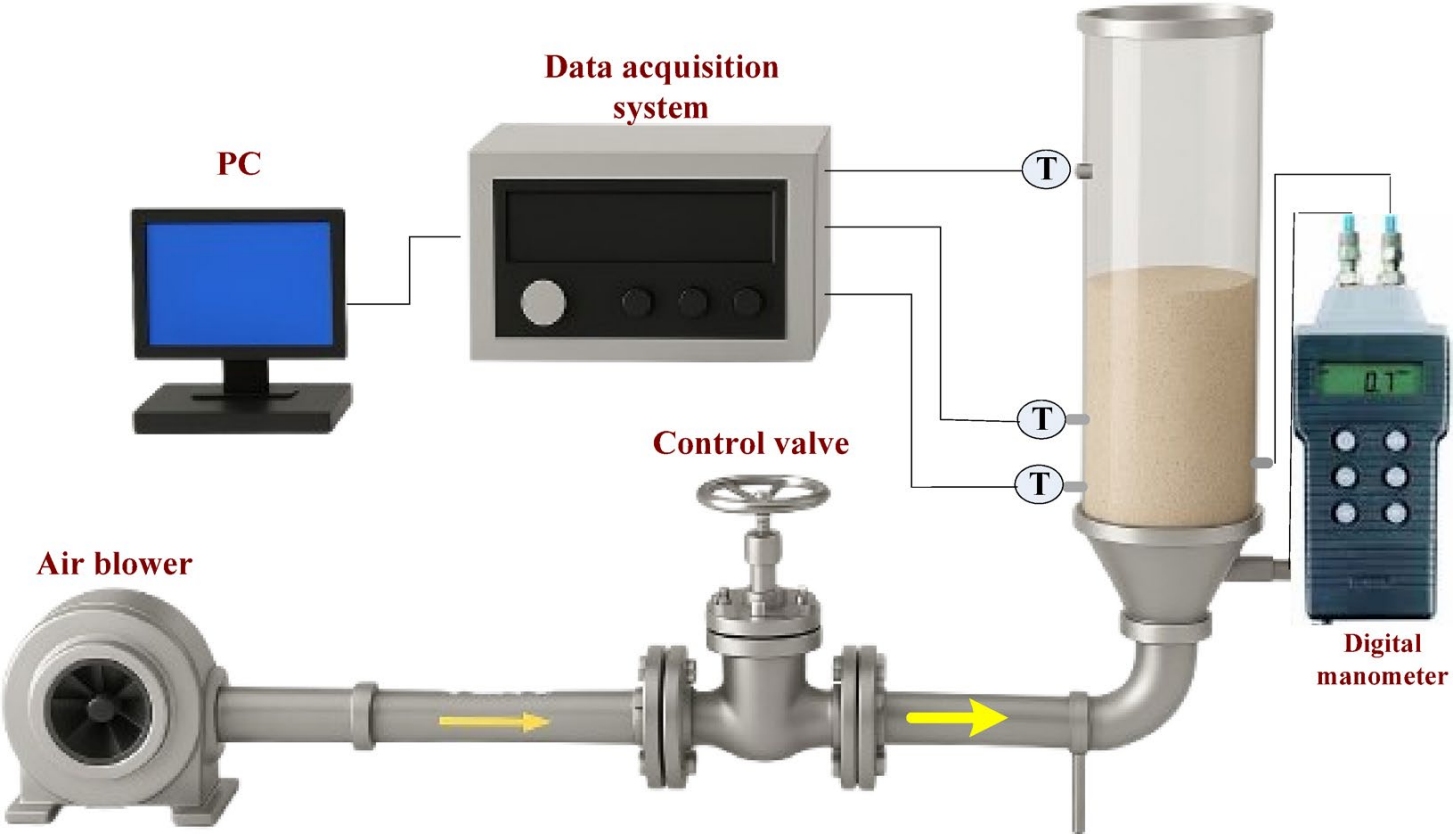
3D representation of the experimental set-up used in this study.

**Fig. 2:**
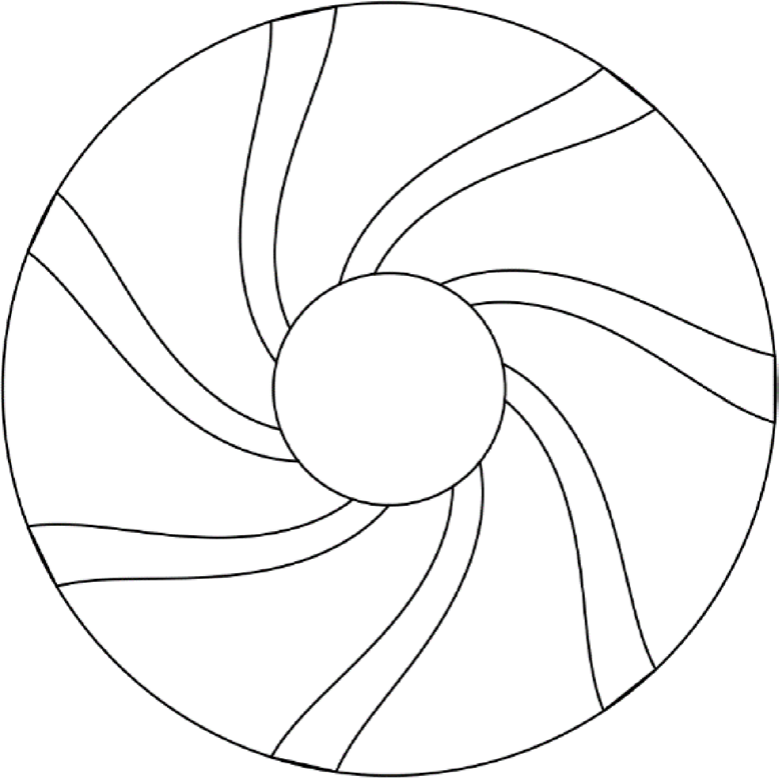
2D schematic of blade distributor used in the experiments.

Figure 1 shows the 3D representation of the experimental setup used in this study. The reactor was made from steel, with a cylindrical shape and a diameter and length of 11 cm and 60 cm, respectively. The fluidized air enters the reactor from the bottom using an air blower through a pipe connected to a control valve to adjust the inlet air to the desired value, while its velocity was measured using a calibrated orifice meter connected to a U-tube manometer. A tubular heater with a diameter and length of 1 cm and 200 cm, respectively, and a total power of 2 kW was selected to heat the bed using heated inlet air. The reactor was insulated using fiberglass insulation to minimize heat losses. The bed temperature was kept at values lower than 400 °C across different air velocities to avoid the radiation heat transfer effect. The annular-blade distributor used in this study, as shown in Fig. 2, consists of 7 blades fixed at an angle of 45 ° to the horizontal, placed at a distance of 1.5 cm on the distributor circumference. The blade was curved and had a trapezoidal cross-sectional area with dimensions of 2.2 × 3.2 cm and was welded to a rotating circular hub with a diameter of 4.2 cm and a height of 2 cm. The rotating distributor used in this study differs from other techniques used to introduce tangential momentum, such as multi-jet or inclined-slot distributors. The mechanical rotation of the distributor actively induces tangential velocity to the flow, which generates a controllable swirl flow adjusted by the distributor’s speed. The curved and trapezoidal cross-section of the blades improves the transfer of momentum in both tangential and axial directions. Additionally, this aerodynamically shaped design decreases pressure losses and flow separation along with increasing the tangential acceleration of inlet air, which enhances both particle mixing and maximum swirling length. The inlet air velocity used in this study ranged from 1.5 m/s to 3.8 m/s, which covers the entire operational range from minimum fluidization up to above the minimum swirling velocity. This range determines the main flow regions, mainly bubbling, turbulent, and developed swirling fluidization states, allowing for the estimation performance enhancement through dynamic conditions.

### Measurements and calculations

Different measurements were conducted to estimate the heat transfer coefficient and bed pressure drop, including measuring the inlet velocity, pressure drop, surface and bed temperatures, and the rotating speed of the distributor. The inlet air velocity was measured using a calibrated orifice, while a digital manometer (Comark C9503/IS, The Comark Company/UK) was used to measure the pressure drop at different heights of 0.01 m and 0.49 m above the air inlet. The rotational speed of the distributor was adjusted by controlling the inlet voltage via DC power supply instrument. The rotational speed of the distributor was measured by a digital tachometer (DT-2234B, CEM Instruments/China).

The temperature measurements were achieved using K-type thermocouples connected to a data logger (Midi Logger GL820, Graphtec Corporation/Japan). Both bed and reactor wall temperatures were measured at different radial and axial positions.

The local heat transfer coefficient was calculated using the following equation:

1 $$h=\frac{Q}{{A}_{s}({T}_{b}-{T}_{w})}$$

where *T*_*b*_ and *T*_*w*_ are the time-averaged bed and wall temperatures, respectively. *A*_*s*_ is the heat transfer surface area and *Q* is the input heat transfer. The heat transfer coefficient was calculated at three bed heights (Z) of 0.035, 0.07, and 0.095 m and at four different radial positions (r/D) of 0.15, 0.4, 0.7, and 0.95 at Z = 0.035 m and at different axial positions. The experiments were repeated three times at each inlet velocity, and each experiment took about 30 min, measuring temperatures every five minutes. The averaged values of the measured parameters were used in the calculations and the analysis of the results.

The uncertainty of measurements was estimated using the Kline and McClintock method^23^ for bed pressure drop and the heat transfer coefficient. The general form of uncertainty (W_R_) is given by.

2 $$W_{R} = \left[ {\left( {\frac{{\partial R}}{{\partial x_{1} }}w_{1} } \right)^{2} + \left( {\frac{{\partial R}}{{\partial x_{2} }}w_{2} } \right)^{2} + \cdots + \left( {\frac{{\partial R}}{{\partial x_{n} }}w_{n} } \right)^{2} } \right]^{{0.5}}$$

Where *R* is the measured value which is a function of ‘*n’* independent variables *x*_*1*_, *x*_*2*_, *…*,* x*_*n*_.

The pressure drop of the bed and distributor was measured using a digital manometer in mm of water. The uncertainty of the pressure drop was calculated using the following equation:

3 $$\Delta P = \rho g\Delta H_{{manometer}}$$

4 $$\frac{{\partial (\Delta P)}}{{\Delta P}} = \left[ {\left( {\frac{{\left( {\frac{{\partial \left( {\Delta P} \right)}}{{\partial (\Delta H)}}} \right)}}{{\Delta P}}w_{H} } \right)^{2} } \right]^{{0.5}} = \frac{{w_{H} }}{{\Delta H}}$$

where ∆H = 0.003 ± 0.45 mm.

As given by Eq. (1), the heat transfer coefficient (h) is a function of many parameters, including the input power (*Q = VI*), the temperature difference *(∆T = T*_*s*_*-T*_*b*_), and the surface area (*As =* πDL). The uncertainty of the heat transfer coefficient can be determined using the following equation:

5 $$\frac{{Wh}}{h} = \left[ {\left( {\frac{{w_{E} }}{E}} \right)^{2} + \left( {\frac{{w_{\Delta } }}{{\Delta T}}} \right)^{2} + \left( {\frac{{w_{{A_{s} }} }}{{A_{s} }}} \right)^{2} } \right]^{{0.5}}$$

The above Kline and McClintock approach was only used to calculate random uncertainty, ignoring the effect of systematic errors. Systematic errors may result from calibration bias, thermocouple drift, and insulation non-uniformities. These contributions were combined with random uncertainties to provide a more comprehensive evaluation of the total error of measurement. Table 2 summarizes the specifications of each measuring instrument, including accuracy, random error, and combined standard uncertainty (${U}_{C}$), which integrates systematic and random errors and is calculated using Eq. 6.

6 $${U}_{C}=\sqrt{{\left(Systematicerror\right)}^{2}}+\sqrt{{\left(Randomerror\right)}^{2}}$$

Considering both random and systematic errors the uncertainty of heat transfer coefficient and pressure drop was ± 3.48% and ± 6.37%, respectively.

**Table 2:** The specification of different measuring instruments with random error.

Instrument	Range	Accuracy (systematic error)	Random error (repeatability)	Combined standard uncertainty
Orifice meter	0–500 mm H₂O	± 1% of full scale (0.5 mm)	± 0.25 mm	± 5.0 mm H₂O (~ 1.0%)
Digital manometer	0 - ±350 mbar	± 0.2% of full scale	± 0.5 mbar	± 1.49 mbar
K-Type thermocouples	0–1100 °C	± 0.2 °C	± 0.1 °C	± 0.22 °C
Data logger	-	± 0.1% of full scale	± 0.05% of full scale	± 0.112%
Digital Tachometer	0–99,999 rpm	± 0.05% ±1 digit	± 0.5 rpm	~±1.7%

### Artificial neural network and PSO model

The artificial neural network (ANN) is an essential machine learning technique employed to tackle the input and output factors of the system and to model the learning and decision-making processes of natural systems. The ANN contains various interconnected artificial nodes (neurons), organized into three main layers. The first layer is the input layer, which contains the input variables; the second layer is the hidden layer, which extracts and processes the main features; and the third layer is the output layer, which creates the final predictions. The ANN depends on the interconnected nodes linked across adjustable weights, which are treated as the memory of the network. These weights enable the ANN to achieve important tasks, including function approximation, pattern recognition, learning, and data generalization. The two critical parameters of the ANN are the biases and weights. The ANN biases introduce an extra degree of freedom by adjusting the threshold of activation functions, promoting the representational capacity of the network. While weights control the magnitude and direction of input signals^24^. The structure of the ANN is estimated depending on the summation of the output of each layer with biases and transferring them by transfer functions to enter the next layer. The backpropagation mechanism is activated when there is a discrepancy between the predicted and target values; this mechanism determines the error between the actual and predicted values and propagates it backward across the network to iteratively adjust the weights and biases. This optimization process reduces the error by gradually enhancing the parameters of the network until the error meets a certain criterion or converges through a predefined tolerance.

**Fig. 3:**
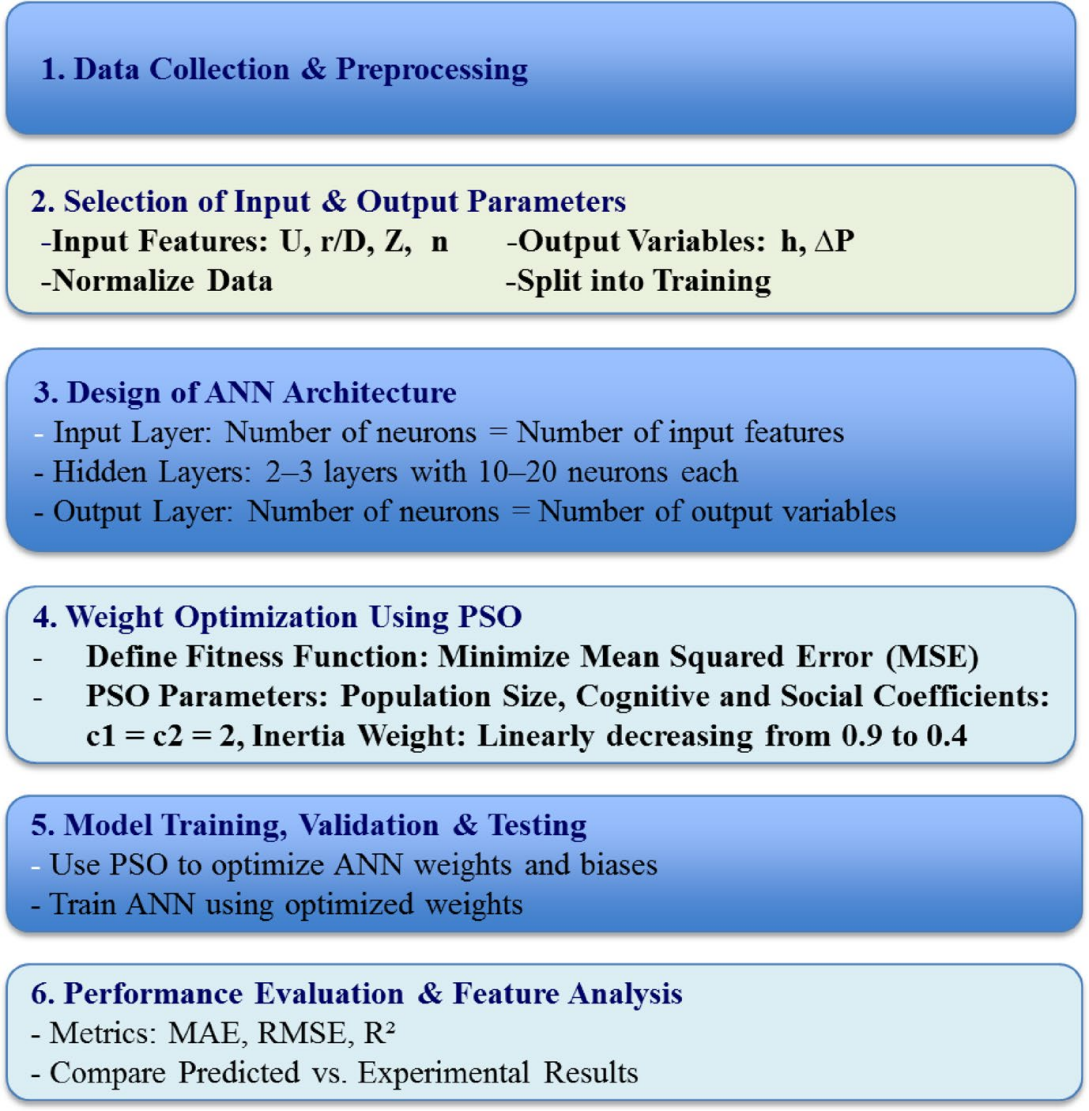
The AI model development flowchart using ANN–PSO.

The structured multi-stage approach used to develop the AI model is shown in Fig. 3. The experimental dataset, including operating parameters along with measured outputs, was collected and normalized to achieve consistent scaling and minimize training bias. The dataset used to generate the AI model was composed of 90 data points, split into training (70%), validation (15%), and testing (15%). Data points used in this study were extracted from the experiments at different operating conditions. The ANN was configured with input, hidden, and output layers, and then coupled with the PSO algorithm to avoid local minima during model training and improve the accuracy of predictions. The ANN biases and initial weights were optimized by the PSO by modeling a swarm of candidate solutions. After the model was trained, the ANN–PSO model was validated using validation and test sets. The ANN–PSO hybrid model was carried out and executed using Google Colab. This platform was selected to achieve reproducibility, accessibility, and simplicity of sharing without the need for specialized software installations. The modeling process was carried out using Python 3.10 through the standard runtime environment of Colab. Different libraries were used, mainly TensorFlow 2.13.0 for developing and training the ANN architecture, Scikit-learn 1.3.0 for data splitting and normalization, and Pandas 2.0.3 and NumPy 1.24.3 for numerical operations and data preprocessing. The error metrics, including mean absolute error (MAE), root mean squared error (RMSE), and regression coefficient (R²) were used to assess the performance of the ANN. The value of R² close to 1 indicates better predictive performance and higher accuracy of the AI model, whereas a low value of RMSE and MSE signifies a lower error between the AI model results and target values. Table 3 provides the detailed parameters of the ANN-PSO model.

**Table 3:** Settings of the ANN-PSO model parameter and model variables.

Parameters	Range	Units
Input variables - Inlet air velocity (U) -Distributor rotational speed (N) -Normalized radial position (r/D) -Axial height (Z)	1.435–2.712 205–924 0.1–0.9 0.01–0.1	m/s rpm - m
Output variables - Heat transfer coefficient (h) -Bed pressure drop (ΔP)	300–950 80–150	W/m^2^ Pa
Number of input layer nodes	4	
Number of hidden layer nodes	8–12	
Number of output layer nodes	2	
Performance function	Mean Squared Error (MSE)	
Backprop weight/bias learning function	-Gradient descent with momentum -Levenberg–Marquardt	
Training function	PSO algorithm	

## Results and discussion

### Comparison between rotating and fixed blade distributors

**Fig. 4:**
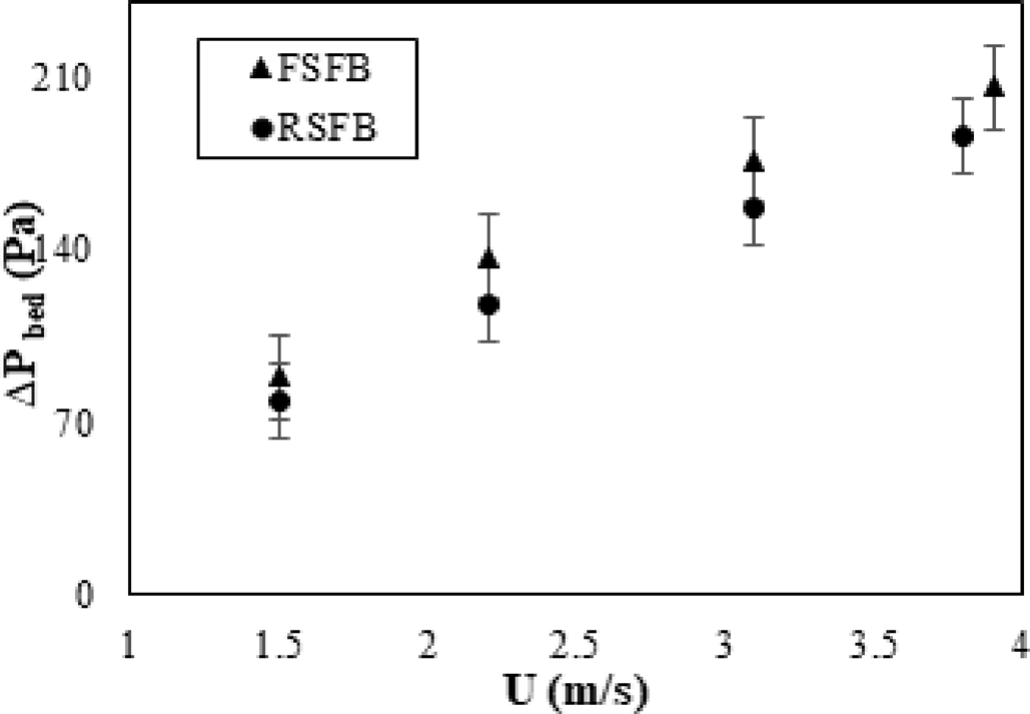
Effect of inlet air velocity on the bed pressure drop for two distributors.

In order to evaluate the effect of a rotating distributor on the operation of SFBR, the obtained results of the rotating distributor were compared to those of fixed distributors (the same distributor with rotating speed of zero). The bed pressure drop is an important characteristic parameter that can indicate the energy consumption of the system. In SFBR, the swirl motion of particles causes an additional downward centrifugal force besides the particle weight, and this force increases as inlet gas velocity increases. As shown in Fig. 4, the pressure drop of the two distributors increased with increasing inlet air velocity; this is expected because the increase in inlet velocity results in increased resistance across the bed due to increasing turbulence and particle motions. Compared to the fixed distributor, the rotating distributor consistently exhibited a lower bed pressure drop, with a decrease in pressure drop of 20% or more through the tested range of inlet velocities. The decrease in pressure drop observed with the rotating distributor may be due to the produced swirling motion along with the rotational effect that creates the tangential velocity, increasing the uniformity of particle distribution and decreasing slugging and channeling impacts. The swirling motion also promotes the mixing of solid particles and decreases elutriation, further decreasing the bed pressure drop. The reduction in pressure drop can directly contribute to a decrease in the energy consumption of the system, resulting in operational cost savings, especially in large-scale conversional systems.

**Fig. 5:**
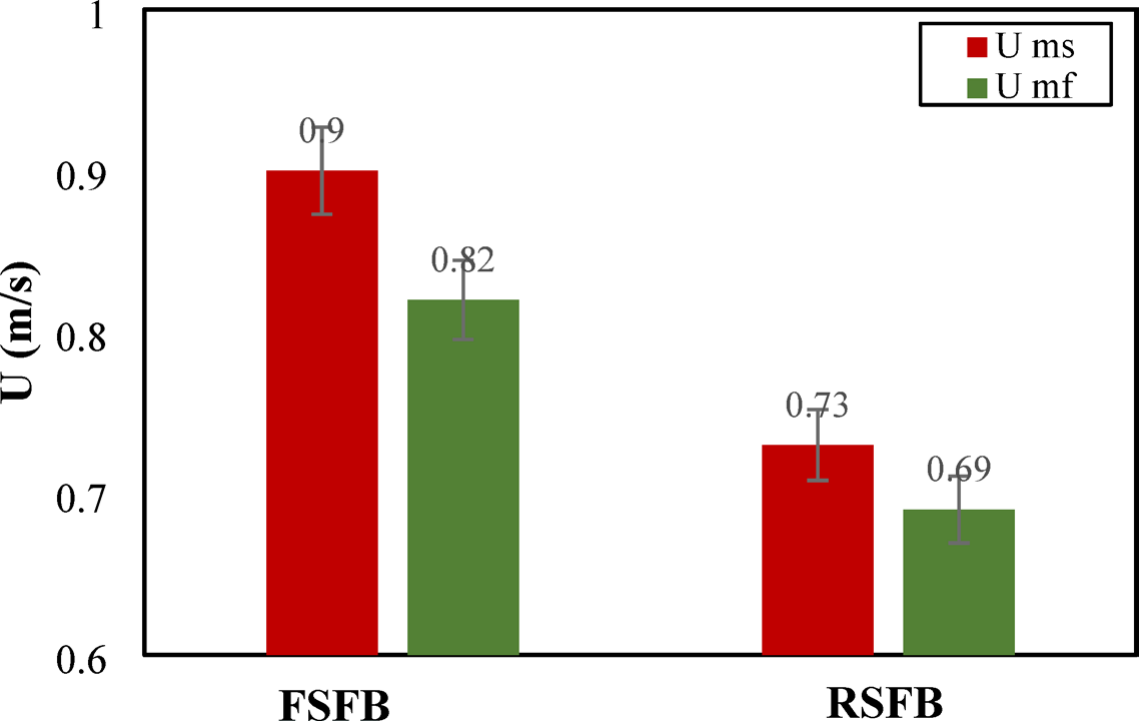
The minimum fluidizing and swirling velocities for two distributors.

Other parameters that can describe the bed flow dynamics are the minimum and swirling velocities. Figure 5 shows the minimum fluidization and minimum swirling velocities for fixed and rotating distributors. The figure shows that the minimum swirling velocity significantly decreases with the rotating distributor. The decrease in minimum swirling velocity confirms the improved swirl intensity and mixing of solid particles produced by the rotational motion, requiring a lower tangential velocity to maintain the swirling state. Additionally, the rotating distributor shows a lower minimum fluidizing velocity required to fluidize the bed. The reduction in this velocity suggests enhanced fluidization quality by decreasing channeling impacts and improving gas-solid contact. The decrease in minimum fluidizing and swirling velocities confirms the efficient performance of the rotating distributor in achieving fluidization conditions and stable swirling at reduced air velocities. This also emphasizes the efficient operation of the rotating distributor resulting in enhanced scalability for large-scale application and energy savings.

**Fig. 6:**
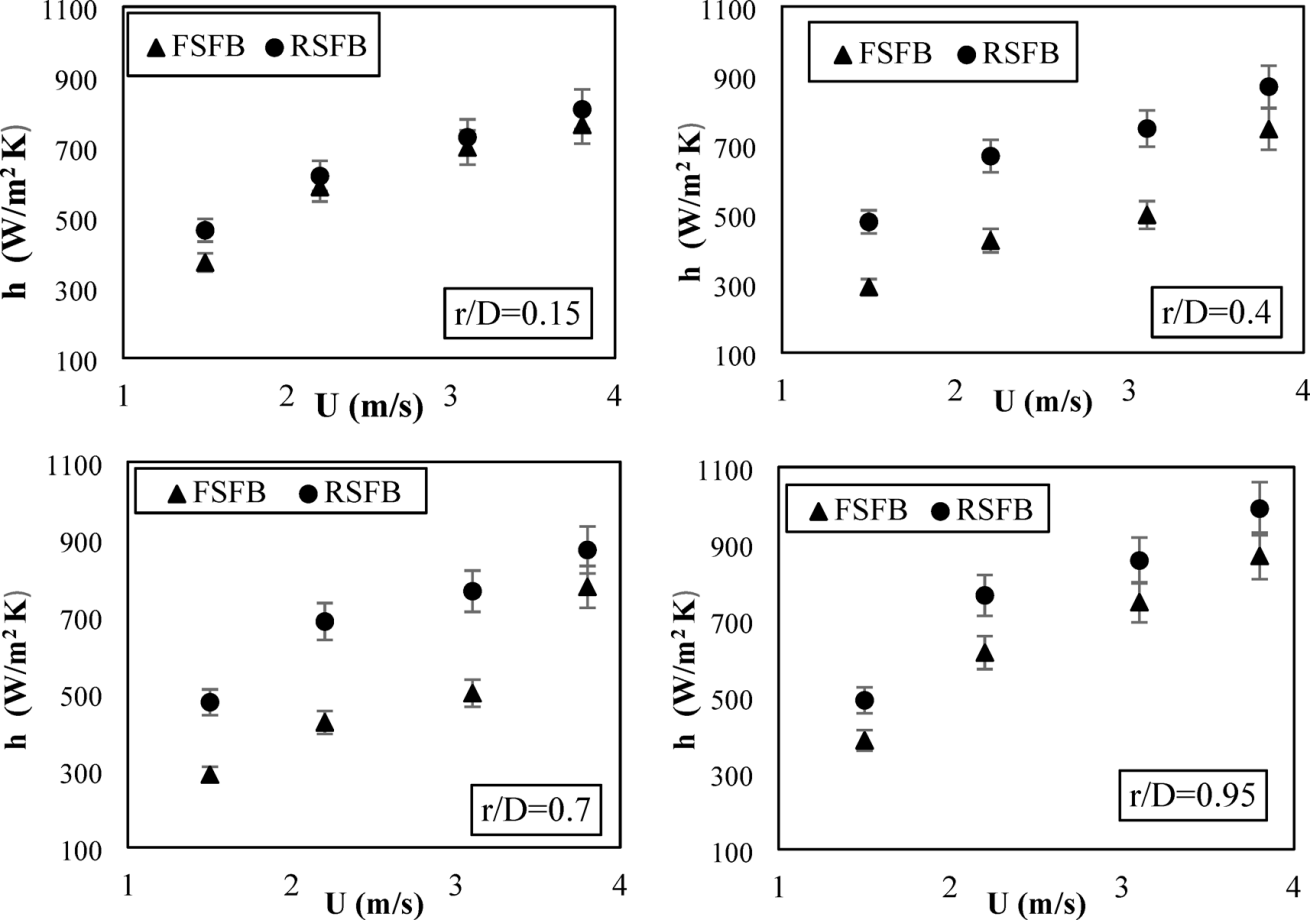
Effect of inlet air velocity on the local heat transfer coefficient at different radial positions at Z = 0.035 m for two distributors.

Figure 6 shows the time-averaged local heat transfer coefficient for both rotating and fixed distributors at different radial positions (r/D) and inlet velocities at a height of 0.035 m. The figure indicates that the heat transfer coefficient increased with increasing air inlet velocity for both distributors at all radial positions. An increase in the inlet velocity improved turbulence and particle mixing, resulting in an enhanced heat transfer process. At all radial positions, the heat transfer coefficient of a rotating distributor was higher than that of a fixed distributor throughout all inlet velocities. At higher inlet velocities, the variance in heat transfer coefficient for the two distributors showed a more pronounced difference, illustrating that the advantages of the rotating distributor are most clear under more dynamic flow conditions. The superiority of the rotating distributor is due to its capability of producing a higher swirling motion, enhancing the mixing of the particles, decreasing channeling, and promoting gas-solid contact. The rotational motion increases tangential velocity, leading to good heat transfer, especially at higher radial positions where swirling influence is most evident.

**Fig. 7:**
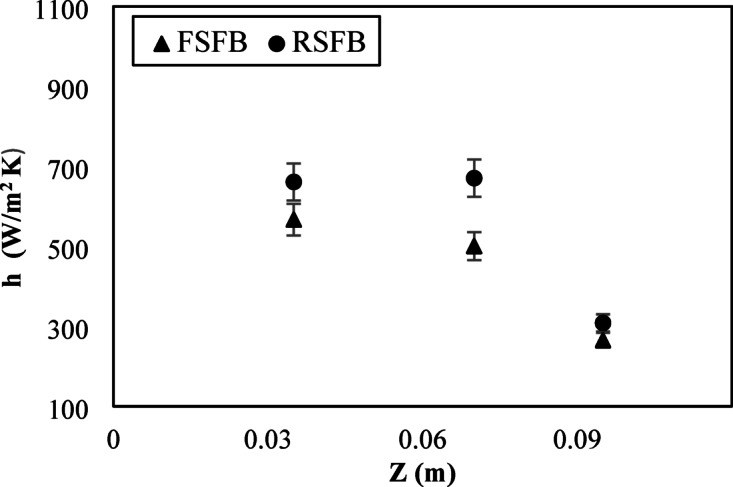
Heat transfer coefficient axial distribution at r/D = 0.4 and U = 3.1 m/s for two distributors.

**Fig. 8:**
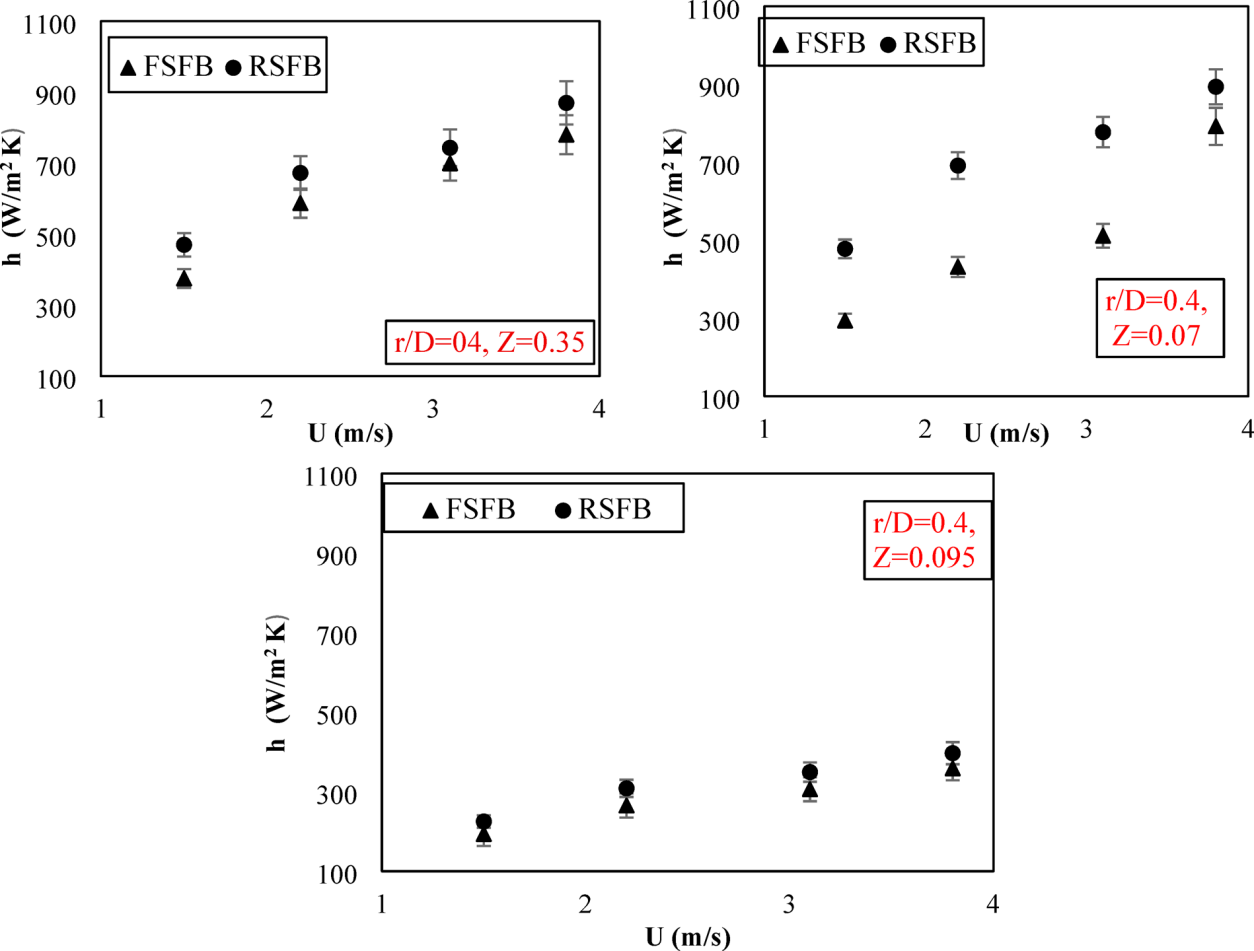
Effect of inlet air velocity on the local heat transfer coefficient at different axial heights.

Figure 7 shows the axial distribution of the heat transfer coefficient at (r/D) = 0.4 and the velocity of 3.1 m/s for two distributors at three different heights of 0.035, 0.07, and 0.095 m. For the two distributors, the heat transfer coefficient was highest at the lower parts near the air inlet. At the lower height, the bed particles are in a fully swirling state due to the tangential component of velocity, which results in excellent gas-particle mixing and an increase in heat transfer. For a fixed blade distributor, the heat transfer coefficient increased at lower heights where the tangential velocity produced by the distributor blades was high. With increasing bed height above 0.035 m, the tangential velocity gradually reduced compared to the axial velocity of the inlet air, resulting in minimizing the swirl effect and leading to a reduction in the heat transfer process. For a rotating blade distributor, the heat transfer coefficient gradually increased from the lower heights until a height of 0.07 m and then decreased. This demonstrated that the rotating blade distributor increased the maximum swirl length, hence increasing the tangential velocity of bed particles, which intensified the mixing and heat transfer process at higher positions compared to the fixed blade distributor. The heat transfer coefficient of the rotating blade distributor was higher than that of the fixed blade distributor for all inlet air velocities, as shown in Fig. 8. Increasing the maximum swirling length also gives the rotating blade distributor an extra advantage over the fixed blade distributor.

### Effect of rotational speed of the distributor

**Fig. 9:**
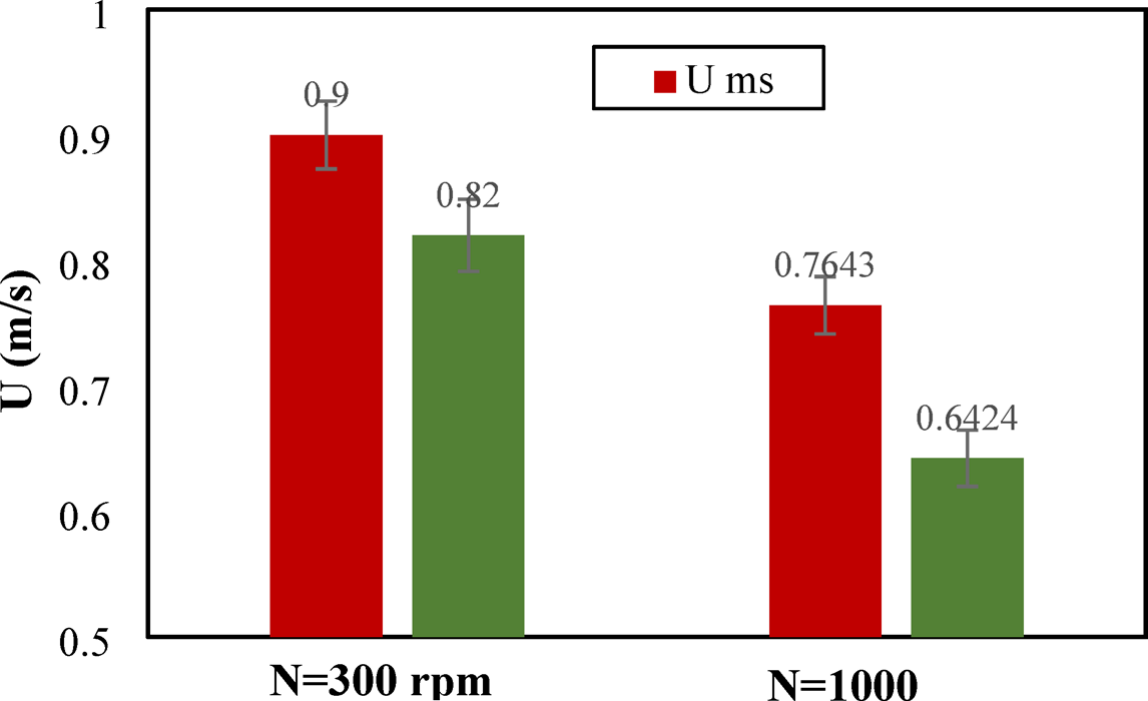
The experimental values of minimum fluidization and swirling velocities for different distributor rotational speed.

**Fig. 10:**
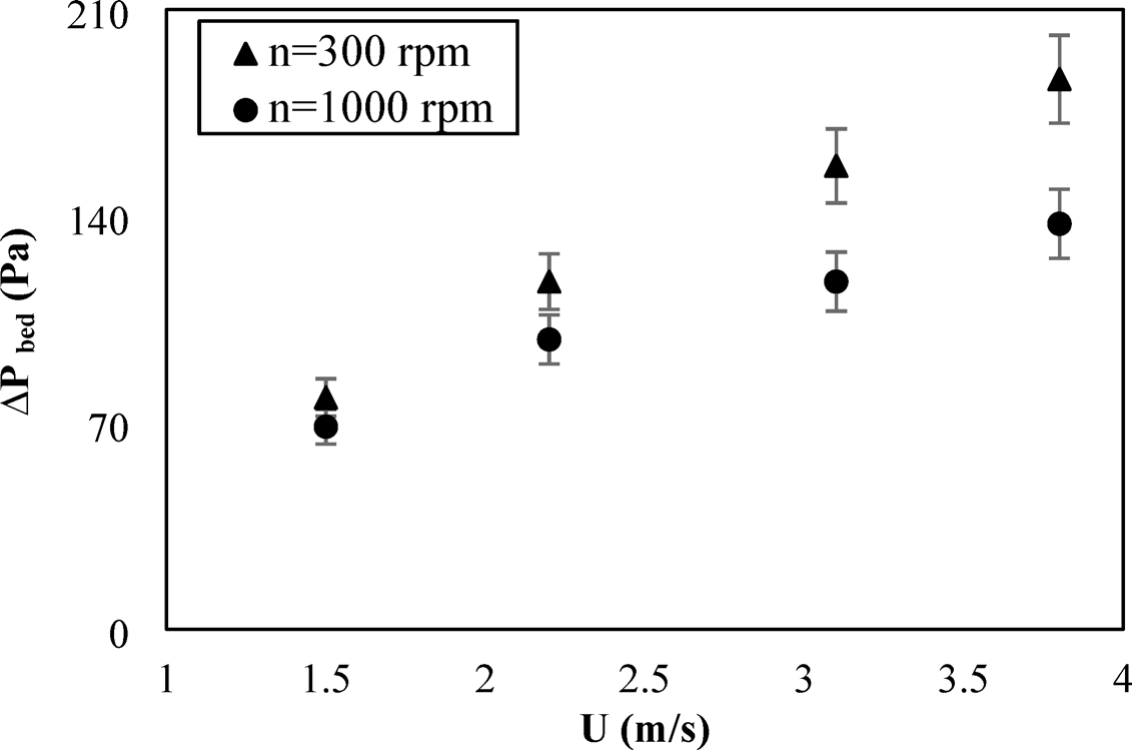
Effect of rotational speeds on the bed pressure drop.

The effect of increasing distributor rotational speed on reactor performance was also studied. The rotational speed of the distributor was increased from 300 rpm to 1000 rpm. Figures 9 and 10 illustrate the effect of distributor rotational speed on fluidization and swirling, as well as the bed pressure drop, respectively. The figures indicated that the fluidization and swirling velocities, as well as the bed pressure drop, decreased with increasing rotational speed. Increasing the rotational speed increased the tangential velocity imparted to the flow by the distributor. The reduction in the minimum fluidization velocity with increasing rotational speed agrees with the findings of other studies [26–28]. However, the reduction in bed pressure drop with increasing rotational speed disagrees with the other studies [26–28] that used a conventional distributor. This is attributed to the conventional distributor’s predominant effect of inlet air axial velocity at higher air flows, which opposes the tangential velocity generated by the rotation of the distributor. This effect was removed when a blade-rotating distributor was used, which resulted in a reduction in bed pressure drop with an increased rotational speed of the distributor.

It should be taken into account that, increasing rotational speed decreased fluidization and swirling velocities, as well as bed pressure drop, thereby decreasing the energy consumed by the system. However, increasing rotational speed also results in an increase in the electrical energy required for the system. Therefore, there should be a limit to the increase in the rotational speed of the distributor.

**Fig. 11:**
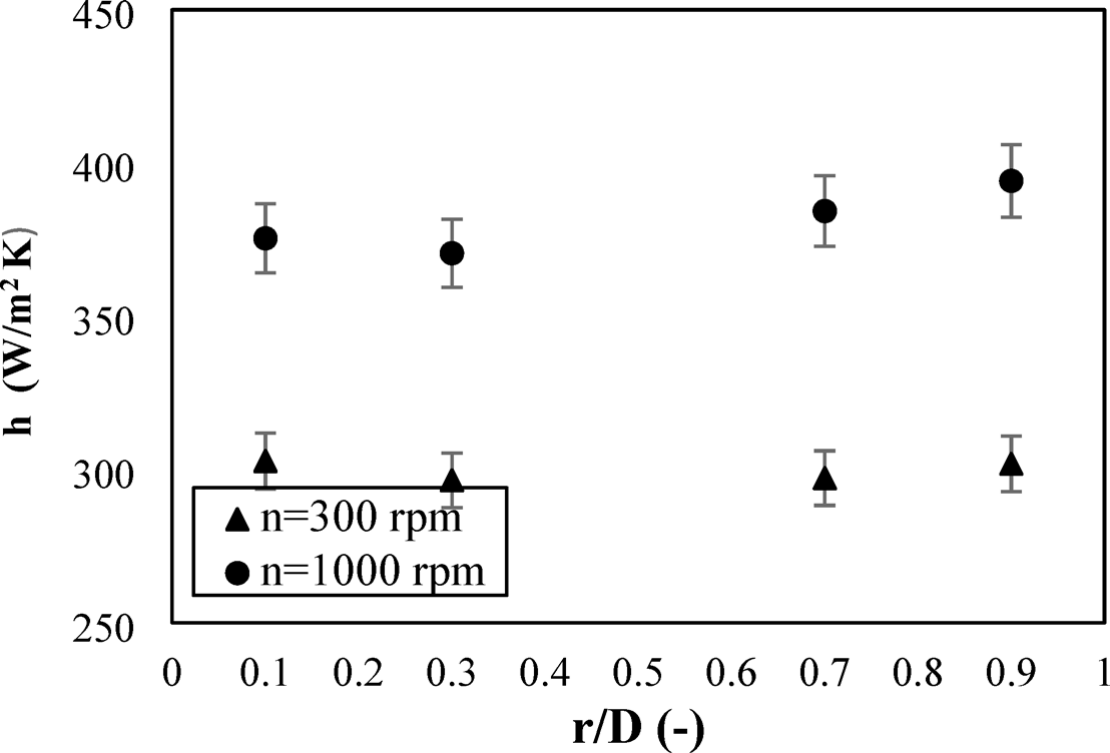
Radial distribution of heat transfer coefficients at different radial positions at Z = 0.035 m and U = 1.5 m/s.

The effect of rotational speed on the heat transfer process is shown in Fig. 11. The heat transfer coefficient was estimated at different radial positions and a height of 0.035 m at an inlet velocity of 1.5 m/s. Increasing the rotational speed increases the heat transfer coefficient at all radial positions. This is expected; increasing the speed of the distributor increases the tangential velocity and hence increases the momentum in the radial direction. Additionally, it increases the maximum swirling length. All of these factors enhance the mixing process inside the reactor and increase the turbulent motion of solid particles within the reactor. This results in further improvement of the heat transfer from the bed to the walls.

### AI results

**Fig. 12:**
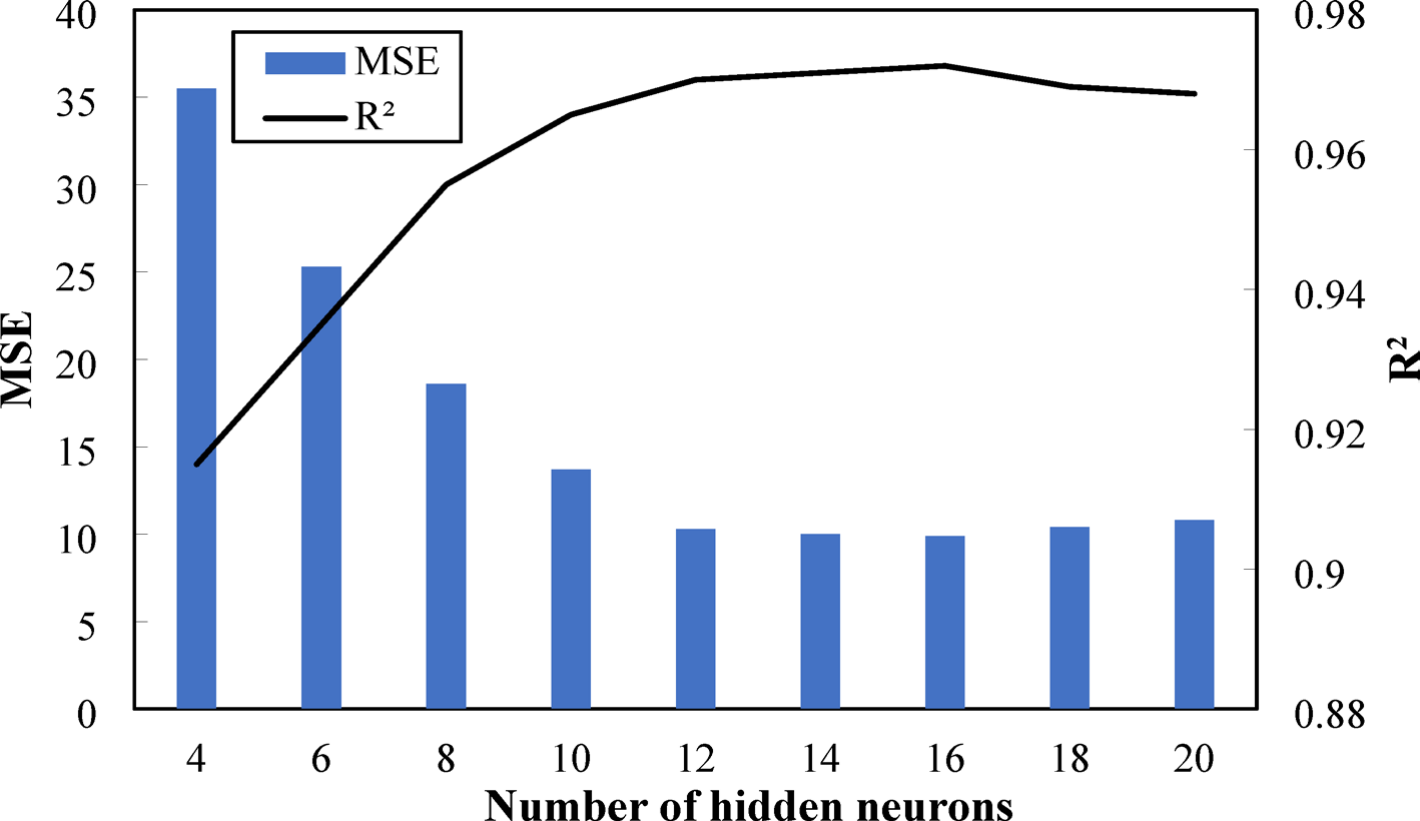
Variation of MSE and R^2^ with the number of hidden neurons.

The estimation of the number of neurons in each layer and the optimum number of hidden layers is essential in ANN architecture design. The variation of MSE and R^2^ with the number of hidden neurons is illustrated in Fig. 12. The performance of the AI model was estimated using two mean metrics: MSE, which indicates the average deviation between predicted and actual values, and R^2^, which highlights the goodness of fit between predicted and experimental results. The figure elucidates that the R^2^ of the model gradually increased with the increase in the number of hidden neurons, reaching a maximum value of 0.972 at 16 neurons, then decreased. This emphasizes the enhanced accuracy of the model with a higher ability to capture the nonlinear relationship between input and output variables. On the contrary, the model’s MSE gradually reduced with increasing the number of neurons, with the lowest value of 9.9 at 16 neurons, indicating a minimal deviation between the predicted and actual values. This alludes that, the optimum number of neurons in the hidden layer was 16.

**Fig. 13:**
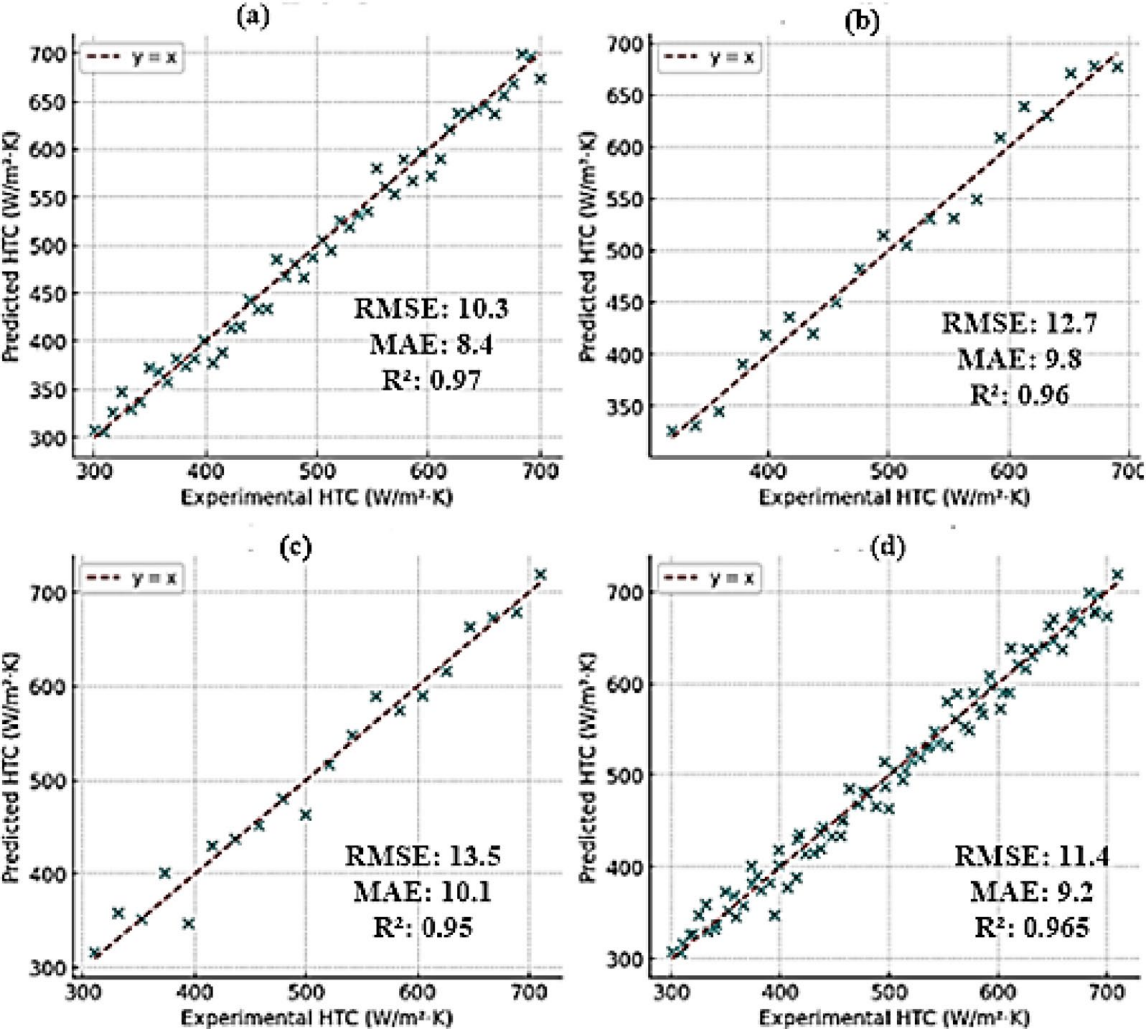
Comparison of predicted and experimental HTC values for (**a**) train set (**b**) validation set (**c**) test set (**d**) all set.

The comparison between the predicted and experimental heat transfer coefficient values for the training, validation, and test datasets is illustrated in Fig. 13. The good agreement of data points across the 45-degree reference line (y = x), emphasizes the high predictive accuracy of the ANN–PSO model. For the training dataset (Fig. 13a), the model yields a high R² of 0.97 and a low RMSE of 10, indicating good learning of the underlying data patterns. The validation set (Fig. 13b) shows strong predictive power with an R² of 0.96, indicating low overfitting and good generalization capability. The test dataset (Fig. 13c) also shows consistent performance with an R² of 0.95, illustrating the ability of the model to reliably predict unseen data. For all data combinations (Fig. 13d), the overall R² is close to 0.97, which further validates the of the ANN–PSO model’s effectiveness and robustness, architecture in modeling complex nonlinear relationships within the swirling fluidized bed reactors. The model results demonstrate the suitability of the proposed hybrid model for real-time prediction and optimization performance of fluidized bed reactors.

**Fig. 14:**
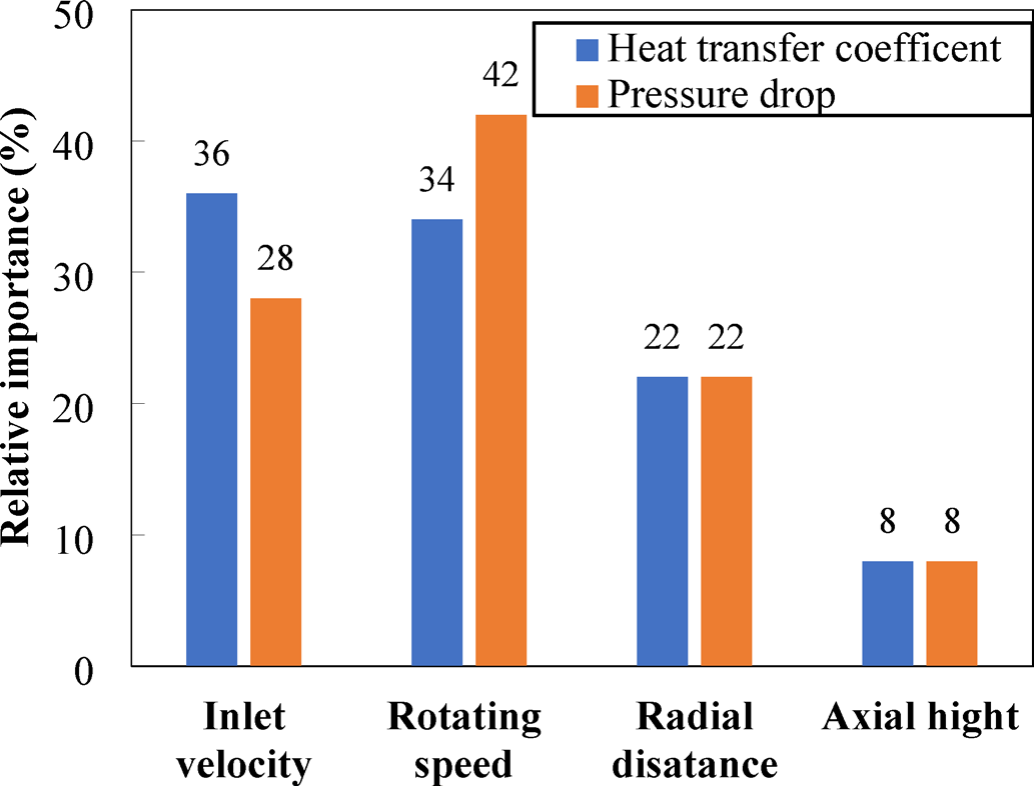
The relative importance of different operation parameters.

In this study, the Garson algorithm [29] is employed to evaluate the connection weights derived from the trained ANN-PSO model to assess the relative importance of each input variable. Figure 14 illustrates the relative importance of each operating parameter on the bed pressure drop and heat transfer coefficient. The inlet air velocity has the most significant importance on heat transfer at 36%, followed by the rotational speed of the distributor at 34%. This suggests the critical roles of these two parameters in improving gas-solid contact and hence promoting convective heat transfer. The radial position (r/D) had a moderate effect (22%), while the axial height (Z) had the minimum impact (8%). This may be attributed to the relatively uniform axial gradient of temperature within the measurement region. In contrast, the rotational speed of the distributor had the strongest influence on the bed pressure drop at 42%, followed by the air inlet gas velocity (28%) and radial position (22%). The higher rotational speeds increased the tangential air momentum, creating enhanced drag and circulation of solid particles, contributing to the elevated pressure losses. The axial height also had a minor impact on pressure drop by 8%, similar to the heat transfer coefficient. These findings validate the ANN model’s internal structure and boost the physical understanding of heat transfer and bed flow characteristics in the swirling fluidized bed reactor. The Garson-derived advantage rankings provide a transparent way to explicate the AI model and estimate predominant operational levers for the optimization of reactor performance. It should indicate that the use of Garson’s algorithm to assess the relative importance analysis should be provided as a statistical measure of input parameter effect instead of direct mechanistic validation. Although the results agreed with known physical trends, such as the strong effect of inlet air velocity and rotational speed on heat transfer, the algorithm does not give causative insights into the implicit solid-gas interactions. Therefore, the interpretability of the ANN–PSO model is limited to determining the participation of the variable, and further coupling with physics-based models, such as CFD models, could be important to fill the gap between the importance of the statistical variable and mechanistic validation.

The developed ANN–PSO hybrid model performance was assessed using standard statistical metrics: root mean square error (RMSE), mean absolute error (MAE), and coefficient of determination (R²), as given in Table 4. For the heat transfer coefficient, the model achieved RMSE, MAE, and R² values of 10.92 W/m²·K, 8.67 W/m²·K, and 0.991, respectively. These values highlight a high accuracy of the model with minimal deviation from experimental results. This confirms a strong ability of the model to capture the underlying physical patterns of heat transfer behavior in the SFBR. Similarly, for the bed pressure drop (ΔPₑ), the model yielded RMSE, MAE, and R² values of 23.73 Pa, 18.91 Pa, and 0.982, respectively, suggesting similarly robust predictive performance of the model. The high values of R² for both target outputs confirm that the ANN–PSO model can describe over 98% of the variance observed in the experimental results. The low values of RMSE and MAE also indicate consistently small prediction errors throughout the dataset. These outcomes validate the reliability and generalization capability of the model in determining the complex nonlinear interactions between input operational parameters and flow behavior in the reactor. The high accuracy of this data-driven model supports its application as a predictive approach for process monitoring, control strategy evolution, and design optimization in gas–solid fluidized bed reactors.

**Table 4:** Error metrics for the AI model.

Metric	Bed Pressure Drop (Pa)	Heat Transfer Coefficient (W/m²·K)
RMSE	23.729	10.915
MAE	18.905	8.674
R^2^	0.982	0.991

**Fig. 15:**
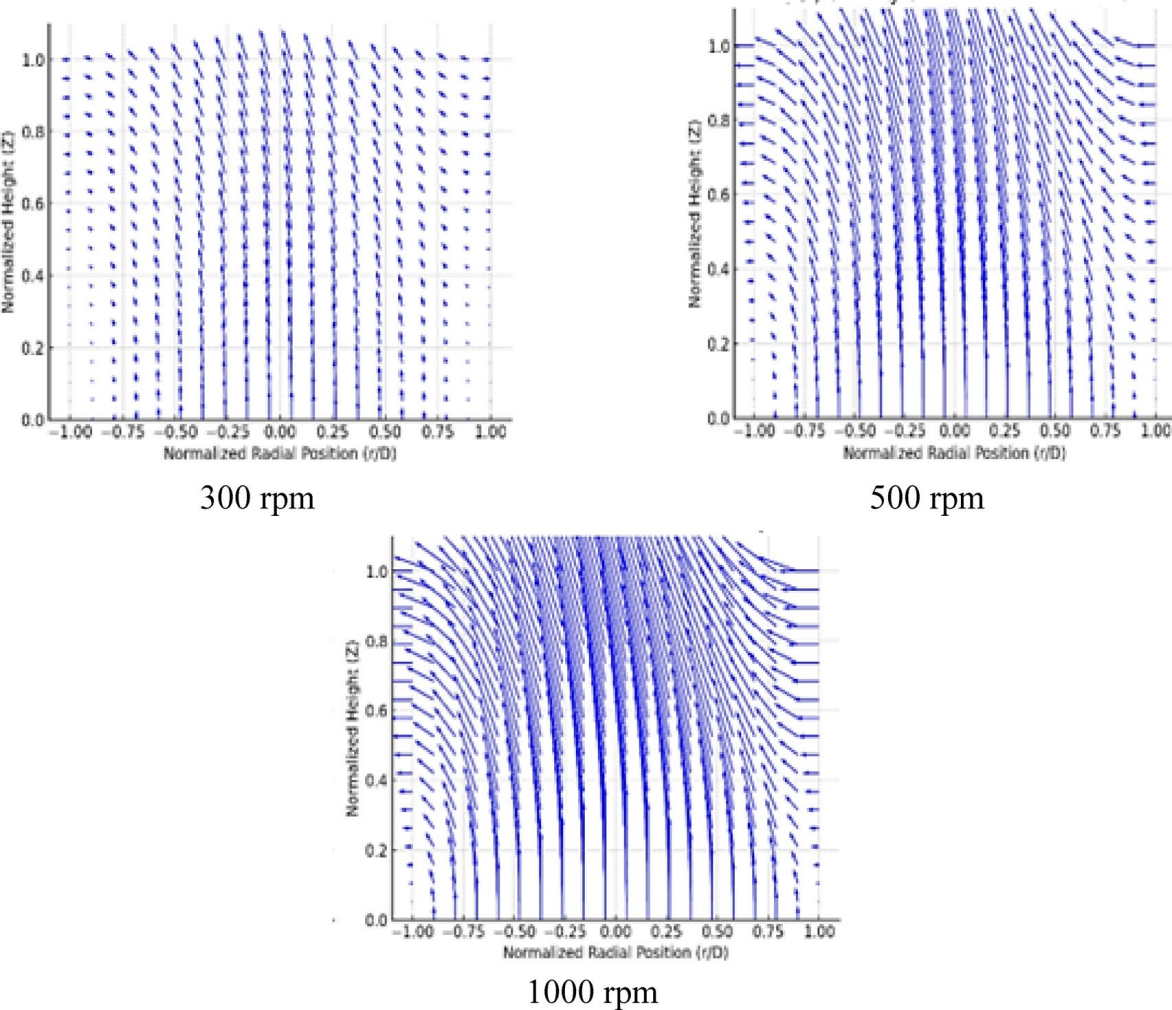
The velocity vector at different distributor rotating speeds.

The velocity vectors in the bed region of a SFBR at three different rotational speeds of 300, 500, and 1000 rpm are displayed in Fig. 15. The prediction results from the ANN–PSO model were used to create the velocity vector fields, capturing radial and axial velocity components through a normalized 2-D spatial grid (r/D, Z). At the lowest rotational speed of 300 rpm, the velocity vector had relatively weak radial motion with moderate axial transport, suggesting a high uniform upward flow and limited swirl generation of the gas-solid mixture. As the rotating speed increases to 500 rpm, a declared swirling pattern appears. Near the reactor centerline, the axial velocity vector becomes more dominant, while at the wall, the radial velocity points inward. This shows that the increase in tangential momentum improves the particle circulation and core-annular flow behavior. At the highest rotating speed of 1000 rpm, the flow pattern becomes increasingly complex, with reinforced radial convergence and localized regions of high axial velocity. This swirling motion characterizes the vortex-improved mixing, where the distributor creates centrifugal forces, that promote a strong inward-deflected radial component near the bed surface, coupled with an upward axial jet in the reactor core. These effects result in enhanced uniform profiles of temperature, fluidization quality, and possibly more effective heat transfer through the bed. The ability of the ANN–PSO model to successfully model these nonlinear spatial flow patterns, emphasizes its ability to generalize the effect of rotational speed on local bed hydrodynamics. Such visualizations offer essential insights into the operational harmony of SFBRs. This can enable optimization of reactor performance across distributor speed modifications and enhance the design of gas-solid contact regions inside reactors.

## Statistical analyses

In this section, two statistical analyses were carried out between experimental measurements and predicted AI model results: one-way analysis of variance (ANOVA) and paired t-test. These analyses were employed to estimate the statistical reliability and accuracy of the ANN-PSO model in predicting and modeling the performance of the reactor. The one-way ANOVA was used to estimate the physical effect of operating parameters on reactor operation. It evaluates whether the average values of the experimental versus predicted datasets differed significantly throughout the full sample. Meanwhile, the paired t-test estimates the potential bias and predictive accuracy of the AI model by comparing its results against experimental findings under the same conditions. It was used to estimate whether there were differences between experimental and predicted values, assuming matched pairs under similar operating conditions. The assumptions used in the ANOVA test include, residual normality, variance’s homogeneity, and independence of data points independence. For the paired t-test, the assumptions include normality of differences between predicted and experimental results and paired observations, as every prediction was achieved under the same input condition of a measured value.

Table 5 gives the values of the ANOVA and t-test analyses for heat transfer and bed pressure drop. The ANOVA results for the heat transfer coefficient showed *p* = 0.924 and F = 0.00007, indicating that the variation under the entire range of parameters was low. The t-test results for heat transfer coefficients show statistically significant impacts with *p* = 0.033 and t = 2.33, which highlight a modest deviation between experimental and predicted values. The ANOVA results (*p* = 0.994 and F ≈ 0.00007) and t-test (*p* = 0.897 and t = 0.13) for bed pressure drop demonstrate good agreement between experimental findings and predicted model results without detection of any statistically significant variation. The difference between p-values from ANOVA and the paired t-test resulted from their distinct analytical objectives and does not elucidate inconsistency. The non-significant ANOVA indicates stable average HTC across rotational speeds, while the significant paired t-test reflects a small but detectable model bias. The two testes provide an accurate view of both reactor behavior and the performance of the ANN model.

**Table 5:** Statistical analyses of the study results.

Parameter	Test type	*p*-value	Test statistic	Significance
Heat transfer coefficient	t-test	0.033	t = 2.33	Significant
Bed pressure drop	t-test	0.897	t = 0.13	Not significant
Heat transfer coefficient	ANOVA	0.924	F = 0.009	Not significant
Bed pressure drop	ANOVA	0.994	F ≈ 0.00007	Not significant

For further confirming the robustness and credibility of the proposed AI model, the performance of the ANN-PSO model was also compared to widely used models, Support Vector Machines (SVM) and Long Short-Term Memory (LSTM) networks, to validate its superiority. The three models were trained on the same dataset (70%), validated (15%), and tested (15%) under the same conditions. The comparison of performance metrics is summarized in Table 6. The findings indicate that the proposed model outperforms the LSTM and SVM models based on higher R^2^ and lower values of RMSE and MAE. This eminent performance is due to the capability of the PSO model to prevent local minima and optimize initial weights, improving prediction accuracy and conversion. Furthermore, the dataset used was composed of steady-state operating conditions, making the model more suitable, compared to LSTM, which is designed for sequential data, and SVM, which struggles with nonlinear complexity. This additional analysis intensifies the generalization capability and reliability of proposed ANN–PSO model.

**Table 6:** The performance metrics of different models.

Model	Output Parameter	RMSE	*R*²	MAE
ANN–PSO	h	10.92	0.991	8.67
	ΔP	23.73	0.982	18.91
LSTM	h	14.31	0.976	11.45
	ΔP	29.85	0.963	23.04
SVM (RBF kernel)	h	18.74	0.952	15.22
	ΔP	35.67	0.941	28.11

To relieve the risk of overfitting that may result from the relatively small dataset used in model development, 5-fold cross-validation was additionally applied. The dataset was split into 5 folds, each containing 18 points. The model was trained on four folds with 72 points and tested on the remaining fold, repeating this process five times. The average RMSE and R² values across folds for the heat transfer coefficient were 12.1 W/m²K and 0.983 ± 0.006, respectively. Meanwhile, the average RMSE and R² values across folds for pressure drop were 26.4 Pa and 0.975 ± 0.008, respectively. These findings affirm that the ANN model exhibited high accuracy across different datasets, with low performance variance, which emphasizes its ability to robustly capture the behavior of the system.

## Implications for engineering applications

**Fig. 16:**
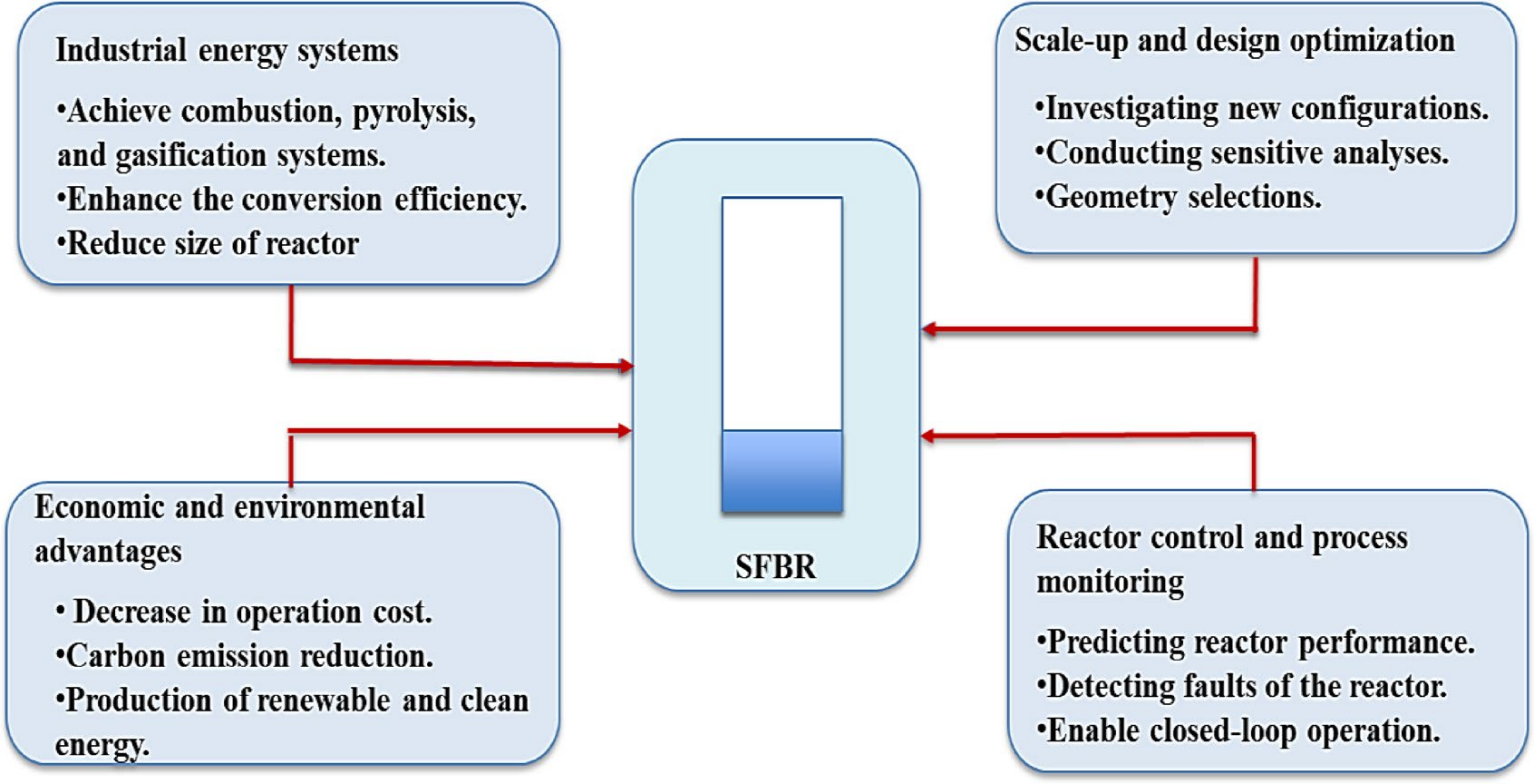
The proposed key domains of practical applications.

The study outcomes integrated experimental results and an AI predictive model, which have a direct relationship to the optimization and potential utilization of SFBRs in real-world energy systems. The improved operation of the reactor due to the utilization of a rotating distributor, as well as, the predictive ability of the ANN–PSO model, can present various ways for practical applications throughout various main domains, which are summarized and shown in Fig. 16. The uniform temperature distribution and high mixing intensity of SFBR increase its utilization in different energy conversion systems such as combustion, pyrolysis, and gasification. The higher increase in the heat transfer coefficient of 56% due to the use of a rotating distributor can promote the thermal conversion of solid fuel, decrease reactor size, and hence costs, extending its use in different industries. The scale-up and design optimization of the reactor can benefit from the obtained results. The model results can provide a scalable new design of the reactor by investigating non-tested configurations without the requirement for expensive and time-consuming experiments. The results also determine the most effective factors impacting the hydrodynamics and heat transfer by conducting different sensitivity analyses along with the selection of geometry parameters such as blade angle and distributor size.

The enhancement in the performance of the reactor with the use of rotating distributor can be translated to an improvement in practical effects in terms of energy saving and cost reduction. The decrease in bed pressure drop by around 20% can directly decrease blower input energy; this reduction can be translated into about 10–12% savings in the total power consumption of system, considering the power required to rotate the distributor, with typical blower capacities (~ 50 kW). The decrease in required power means using smaller blowers with smaller structural support, leading to possible decrease in capital cost. Furthermore, the observed enhancement in the heat transfer coefficient of up to 56% suggests potential for decreased reactor size and heat transfer surface area for the same thermal output, providing potential savings in capital and operational costs in large-scale designs. The reduction in reactor size and blower can also be translated to reduction in carbon emissions, which is considered environmentally advantage. These findings elucidate possible engineering advantages that could support future applications in thermal conversion reactors.

## Conclusion

Swirling fluidized bed reactors are used in various applications such as combustion, pyrolysis, and gasification to solve the problems of conventional FBRs. A blade distributor is used to produce a swirling motion that improves the heat transfer process. For further improvement of the SFBR operation, a rotating blade distributor was used in the present study. This study introduces and validates a novel hybrid approach that combines experimental results with an ANN–PSO to model the performance of the SFBR coupled with a rotating distributor. The bed-to-wall heat transfer and bed hydrodynamics for the rotating blade distributor were investigated experimentally. The main results obtained are given in the following points:

1. The heat transfer coefficient of the rotating blade distributor was always higher than that of a fixed distributor for all different radial positions and all axial heights.
2. The increase in the distributor’s rotating speed from 300 to 1000 rpm improved the heat transfer coefficient from 280 to 438 W/m²·K with an increase of 56%.
3. The ANN–PSO model successfully captured reactor performance with high accuracy (R² > 0.98), and predicted values of HTC and ΔP with average errors below 2%.
4. Relative importance analysis indicated that inlet gas velocity was the most influential parameter on the heat transfer coefficient (36%), followed by distributor rotating speed (34%).
5. Relative importance analysis also confirmed that distributor rotating speed (34%) was the most influential parameter on bed pressure drop (42%) followed by inlet gas velocity (28%).
6. The AI hybrid model enabled the visualization of velocity vector distribution, reinforcing the experimental findings.

### Limitations and future work

This study investigates the heat transfer process and bed flow in a SFBR considering the use of rotating distributor using experimental methods along with an AI model. Although both experimental and AI models successfully measured and predicted the heat transfer and pressure under different operating conditions, some important limitations should be considered. The experiments were carried out under controlled lab conditions without considering the complex environments of real industrial applications. Despite the obtained results significantly emphasizing the advantages of employing a rotating distributor, future work should consider the reactor operation under actual operating conditions, mainly reactors with larger sizes, real feedstocks such as biomass, wider range of particle sizes, and higher temperatures, to achieve both practical use and scalability of the system. Future research should also address the following recommendations that involve limitations:

- Studying the long-term mechanical resistance of the rotating distributor under high-temperatures and higher rotating speeds above 1000 rpm.
- Using larger and more varied datasets, such as different particle sizes and bed geometries, to improve the transferability and robustness of the AI model.
- The AI results should be validated with advanced diagnostics such as thermal imaging or particle image velocimetry to bridge experimental and computational gaps.
- Integration AI approaches with CFD diagnostics or physics-informed machine learning models should be used to fill the gap between the statistical importance of input variables and mechanistic validation.
- Using lifecycle and techno-economic analyses to optimize the rotational speed of the distributor for practical applications.

## Data Availability

The data that support the results of this study are not openly available due institutional and intellectual-property restrictions associated with the research implementation and its future extension.
